# Overcoming barriers and understanding the psychological impact of timely pig euthanasia on Spanish-speaking swine caretakers in the United States

**DOI:** 10.3389/fvets.2024.1505531

**Published:** 2025-01-20

**Authors:** Pablo Lamino, Renzo Ceme Vinces, Nancy F. Acevedo León, Amy Boren-Alpízar, Marcelo Schmidt, John J. McGlone, Arlene Garcia

**Affiliations:** ^1^Department of Agricultural Education and Communication, University of Florida, Gainesville, FL, United States; ^2^Texas Tech University School of Veterinary Medicine, Amarillo, TX, United States; ^3^Department of Animal and Food Sciences, Texas Tech University, Lubbock, TX, United States; ^4^Department of Agricultural Education and Communications, Texas Tech University, Lubbock, TX, United States

**Keywords:** Pig euthanasia, Comprehensive Swine Industry Assessment (CSIA), North American Free Trade Agreement (NAFTA) visa, Hispanic workforce, pig caretakers

## Abstract

**Introduction:**

This study explores the complex experiences of Spanish-speaking swine caretakers with euthanasia, focusing on mental health, stress, burnout, and the impact of cultural factors.

**Methods:**

We conducted qualitative focus groups with Spanish-speaking swine caretakers from multiple farms, gathering insights from individuals with varying levels of experience and cultural backgrounds. Data were analyzed using thematic analysis to identify key factors influencing the euthanasia process.

**Results:**

The study found that caretaker stress and burnout, exacerbated by the “caring-killing paradox,” significantly affect emotional wellbeing. Factors such as the frequency of euthanasia, level of knowledge and education, and cultural background were identified as influential. Caretakers performing euthanasia frequently showed signs of desensitization, while those with less experience experienced higher emotional distress. Cultural background influenced attitudes and decision-making regarding euthanasia.

**Discussion:**

This research underscores the need for mental health support and culturally sensitive training programs for swine caretakers. The findings align with existing literature on occupational stress and burnout and highlight the importance of comprehensive support systems. Future research should further examine the psychological impact of euthanasia across diverse agricultural settings and develop targeted interventions to support caretakers' mental health and improve animal welfare practices.

## 1 Introduction

Pig euthanasia, as outlined by the National Pork Board [NPB], must be a considerate procedure that minimizes pain and distress for the animal (1). The term “euthanasia” in this context refers to the act of ending the life of an animal to relieve it from pain and suffering. Timely euthanasia is critical when animals are showing signs of disease or distress that cannot be treated. It is important to consider that animals can be euthanized for various reasons, but euthanasia is intended to provide a merciful end. The decision-making process should consider the farm veterinarian's indications, standard operating procedures, the severity of the animal's condition, past treatments, direct observation, and the intended destination of the animal (1, 2, 80).

Identifying and humanely euthanizing compromised pigs requires both observational skills and technical expertise, making euthanasia a multi-step process (3). Veterinary diagnostics are crucial for determining the severity of the animals' conditions and guiding decisions on swine health problems (4). When animals are injured or diseased beyond viability or productivity, and when there is a significant risk of suffering, euthanasia is recommended to prevent further suffering (5, 6). Additionally, past treatments play a role in these decisions; untreated or improperly treated conditions, such as damage to the integument or issues with the respiratory, gastrointestinal, urinary, locomotor, or reproductive systems, may necessitate euthanasia (7). Caretakers are advised to observe the animal for signs of improvement before making a final decision (3, 7). During this observation period, it is crucial to provide appropriate treatment, pain relief, and the use of sick pens to ensure the animal's comfort and wellbeing.

Despite its importance, euthanasia may be delayed or not performed at all due to various factors. Differences in perception among veterinarians, particularly those newer to the profession, can impact euthanasia decisions (8, 9). Gender and ethnic background also influence attitudes, with female Spanish-speaking employees showing more hesitance to euthanize pigs than male Spanish-speaking employees (8, 10). Nonetheless, caretakers generally express concern for animal suffering despite being required to use the least painful euthanasia methods (10–12). According to AVMA (11) guidelines, experience also plays a role; employees with more experience in euthanizing pigs are more willing to perform the task, which is consistent with studies on compassion fatigue in shelter workers (12).

In the swine industry, caretakers are primarily responsible for performing euthanasia, a task that carries significant emotional weight (2, 13). Research has shown that constant exposure to euthanasia procedures can lead to psychological stress and emotional fatigue among caretakers (3, 8). This emotional burden is compounded by challenges related to training, equipment, and the emotional bond between caretakers and pigs, all of which significantly impact the decision-making process (14). Equipment issues, maintenance, and resource constraints can further impede proper euthanasia execution (15). Furthermore, caretakers' experience influences their attitudes toward swine euthanasia, affecting their confidence and empathy toward the animals (17).

In the swine industry, performing euthanasia presents several obstacles, including challenges related to personal boundaries, decision-making, and the availability of training resources (3, 8). These challenges can complicate the process, particularly when it comes to detecting and evaluating pigs in poor condition, managing equipment placement and condition, and handling the disposal of pig carcasses (15). From an animal welfare perspective, delays and inconsistencies in euthanasia practices can result in prolonged suffering, especially when methods are not executed promptly or appropriately. Research highlights the importance of ensuring humane practices, including minimizing pain and distress during euthanasia. For instance, effective containment and gas distribution are critical for CO_2_ euthanasia (81, 82). Similarly, physical methods involving impact to the skull with a solid object or surface are practical for euthanizing piglets on farms but often lack repeatability and accuracy, as success depends on the force exerted by the stockperson (18). These factors can significantly hinder the timely execution of euthanasia, underscoring the complexities involved in ensuring humane practices on farms (3).

Implementing support strategies, such as workshops or management systems, can help caretakers make informed decisions about euthanasia (16). Enhancing the wellbeing of pig caretakers during the euthanasia process not only benefits them but also positively impacts the efficiency and sustainability of the overall operation (13). Sustainability in this context means maintaining a healthy and productive workforce, reducing turnover, and ensuring ethical practices that support welfare (13, 19, 83).

Considering the significant role of the agricultural and food sectors in the U.S. job market, it is noteworthy that over 500,000 agricultural workers are first-generation immigrants of Latino/Hispanic background (20). Given this demographic, exploring cultural differences in caregivers' attitudes, values, and beliefs about performing euthanasia is critical. Moreover, the NAFTA agreement and the Trade NAFTA (TN) status have a direct impact on the workforce in this context, with qualified Canadian and Mexican citizens contributing to nearly five million American jobs (21). Many TN visa holders are classified as agricultural, veterinary, or animal care professionals, making the requirements and regulations of these visas pertinent to this study.

Despite the important role of Spanish-speaking workers in U.S. swine farming, there is still limited research on their perspectives regarding critical animal welfare practices (22, 23), such as timely euthanasia (8, 24). Most existing studies focus on the technical and managerial aspects of euthanasia, often neglecting the workers' viewpoints, which are crucial for effectively implementing these practices (7, 25). Furthermore, cultural, linguistic, and experiential differences among Spanish-speaking caretakers may influence their perceptions and decision-making processes, which remain largely unexplored in the current literature (8, 22, 26, 27). Therefore, given the historical significance and current trends of Spanish-speaking workers within the U.S. swine farming industry, this study seeks to address this gap by investigating the perceptions of Spanish-speaking swine caretakers on the performance of timely euthanasia.

### 1.1 Theoretical framework

In this study, we utilized the theoretical framework of Compassion Fatigue [CF] to explore the experiences of Hispanic swine caretakers, with a particular focus on the challenges they face during pig euthanasia. CF is a recognized phenomenon characterized by “a state of exhaustion and dysfunction biologically, psychologically, and socially as a result of prolonged exposure to compassion stress and all it invokes” [(28), p. 253]; this condition often emerges from continuous exposure to situations requiring compassion, leading to symptoms such as emotional exhaustion, diminished empathy, increased irritability, and higher rates of absenteeism. Roles that involve caregiving and regular encounters with traumatic situations are especially susceptible to CF. Moreover, if left unaddressed, CF can escalate into more serious mental health issues like depression and anxiety, significantly affecting an individual's productivity and overall wellbeing (28–30).

CF is often intertwined with burnout [BO], secondary traumatic stress [STS], and compassion satisfaction [CS]. BO, as initially conceptualized by Maslach et al. (31), is rooted in the interpersonal context of the job, particularly the caregiver-recipient relationship and the values and beliefs related to caring work among care providers. It is commonly defined as “a syndrome of emotional exhaustion, depersonalization, and reduced accomplishments that can occur among individuals who engage in 'people work' of some kind” [(32), p.1]. Over time, the concept of burnout has evolved to encompass the negative effects of all occupations (33).

STS involves experiencing psychological distress due to exposure to the suffering of others (31). CF is a consequence of stress, encompassing work-related and compassion stress. It is important to note that there is a synergistic relationship among various forms of stress, and primary traumatic stress, which results from directly experiencing or witnessing traumatic events, whether at work or in personal life, can significantly contribute to and heighten the risk of compassion fatigue. BO results from failed goal achievement, while STS arises from the inability to rescue or save someone from harm, leading to guilt and distress. When unmediated, BO and STS can lead to CF. In general, CS represents the positive aspect of working with traumatized individuals.

Since the mid-1990s (28, 31, 34), the emotional, cognitive, and physical consequences of providing professional services to trauma victims and survivors have been addressed in the literature, and several conceptual models have been developed to explain them. However, most of the research to date has focused on identifying the prevalence and predictors of CF in a unique occupational group such as nurses (35, 36), therapists (37, 87), community service workers, and healthcare professionals in hospital emergency departments or intensive care units (38). While these studies have shed light on how CF can be addressed, their findings probably cannot be generalized to working populations beyond the healthcare sector. Few studies have extended CF's use for other audiences besides the healthcare sector. However, studies about CF in special education teachers to help understand professional burnout have been reported (39). Additionally, CF has been extended as a theoretical framework for animal-care professionals (40–43), aiming to understand how the constant caring and professional workload can affect the wellbeing of these individuals.

Hispanic pig caretakers are likely to experience compassion fatigue due to the nature of their work, which may involve euthanizing pigs. The farms included in this research were finishing sites and sow farms (8). Caretakers typically worked in barn-specific roles, with most participants employed in farrowing units and smaller proportions in breeding units. The frequency and nature of euthanasia varied significantly by the animals' age and size. Caretakers reported euthanizing up to 2–30 piglets per day, while euthanasia of larger pigs occurred less frequently. These processes often required physical and emotional resilience, as caretakers had to rely on established methods, including blunt force trauma or captive bolt systems (8).

Euthanizing animals can be emotionally distressing, especially if caretakers have empathy and a compassionate connection with the pigs (44, 45). This prolonged exposure to compassion stress, especially in scenarios involving euthanasia, can lead to compassion fatigue. CF may manifest in the form of emotional exhaustion, reduced empathy toward the animals, and an increased risk of absenteeism due to the emotional toll of the work (44, 78, 84). Conversely, some caretakers may struggle to form emotional connections with the animals, especially when faced with the daunting task of depopulating large numbers of pigs to fulfill job requirements (3, 46, 47). This disconnect can create additional stress, compounding the emotional challenges of their role.

The concept of BO, as outlined by Maslach et al. (48), can be applied to Hispanic pig caretakers. BO, rooted in the interpersonal context of the job, pertains to the relationship between the caregivers (the pig caretakers) and the recipients of care (the pigs). It includes emotional exhaustion, depersonalization, and reduced accomplishments. The nature of pig caregiving, especially in situations where euthanasia is involved, can lead to emotional exhaustion and depersonalization, as caretakers may need to distance themselves from the animals to cope with their duties. This can negatively impact their sense of accomplishment and job satisfaction.

In the context of Hispanic pig caretakers, STS would involve the distress they experience during the euthanasia process and the emotional toll it takes on them. Witnessing or being directly involved in euthanizing animals can lead to feelings of guilt and distress, which are characteristic of STS. The distress from euthanasia can interact synergistically with other stressors, contributing to CF.

The purpose of this study was to understand Spanish swine caretakers' perceptions, attitudes, and knowledge of euthanasia. The specific objectives of this work were to: (1) understand the effects of euthanizing pigs on Spanish-speaking caretakers, (2) explore how caretakers' experiences affect performing euthanasia decision-making, and (3) to understand how caretakers could feel more prepared to perform euthanasia.

## 2 Methods

### 2.1 Study design

This work is a continuation of Acevedo et al. (8)'s study. This study was part of a larger research project that used an explanatory sequential mixed methods design, where qualitative data were collected in phase two to explain the quantitative findings (49). Additionally, the quantitative phase was used to identify specific groups of interest for the participant sampling in the qualitative phase (49–51).

A qualitative case study design was employed for the second-phase qualitative portion since the study focused on exploring one or more cases within a bounded system (52). In this research, the identifiable case is the perceptions of Spanish-speaking caretakers regarding pig euthanasia. The study's boundaries were defined by two key factors: location and participant selection criteria. Data collection was conducted in Iowa, providing a specific geographical context for the study. The participant selection criteria further delimited the study's scope.

Qualitative researchers suggest having a philosophical paradigm that frames the study to better guide the research process (53). This lens is used to shape data collection and analysis of this research, ensuring that the study aligns with the chosen approach (53). The social constructivist lens was selected as this study's philosophical paradigm since it aims to explore an event in specific groups' lives and assign meaning to their subjective views. Since this research aimed to understand how participants perceive and make meaning out of their euthanasia experiences, the social constructivist design was selected to frame the study (53).

The study received approval from the Texas Tech University Institutional Review Board [IRB] under the assigned number IRB2019-225. As informed consent was obtained from participants before conducting interviews, the study qualified for exemption. Participant safety was prioritized by maintaining confidentiality and anonymity during the analysis of interview data.

### 2.2 Participant selection

A purposive sample was selected from the caretakers who were part of the quantitative portion of the study to gather insights, comprising individuals with first-hand experience with the phenomenon under investigation (54). To ensure comprehensive representation, the following selection criteria were employed: (1) direct engagement with euthanasia, (2) identify as Hispanic, and (3) willingness to discuss euthanasia-related challenges and opportunities. Additionally, the study excluded individuals under the age of 18.

After completing the initial quantitative phase (8), focus group interviews were conducted with swine caretakers in a rented community space conveniently located near all participating farms. Focus groups were chosen as the primary data collection method because they “create a milieu in which social relations are forged and processes of discussion initiated which are similar to those experienced in everyday settings” [(55), p. 265]. While one-on-one interviews could also provide valuable insights, the communal nature of sensitive topics such as euthanasia warranted a method that could effectively capture the social dynamics underpinning these workplace experiences.

A total of 86 participants from 11 swine farms participated in the study, with each farm forming a distinct focus group. This resulted in 11 focus groups in total. The decision to include 86 participants in 11 focus groups was guided by previous research suggesting that 90% of themes can be identified with as few as six groups (56). Including 11 groups ensured a comprehensive representation of the topic under investigation. Additionally, practical considerations such as the number of accessible farms in the region and participants willing to discuss sensitive issues like euthanasia further shaped the focus groups.

The focus group participants were predominantly young adult males aged between 18 and 35 years. Most participants were from Mexico and held university degrees. About half reported working on their current farm for 13–48 months. Additionally, the majority of caretakers worked in farrowing farms or breeding units. These demographics align closely with industry workforce characteristics (20, 23, 24, 57). Table 1 provides a detailed breakdown of participant demographics.

**Table 1:** Description of the focus group by farm and gender (*N* = 86).

**Focus group (G)**	***N***	**Gender**	
		**Female (*****n*** = **29)**	**Male (*****n*** = **57)**
1. Farm (A)	10	6	4
2. Farm (B)	11	6	5
3. Farm (C)	7	0	7
4. Farm (D)	6	2	4
5. Farm (E)	9	4	5
6. Farm (F)	9	0	9
7. Farm (G)	6	2	4
8. Farm (H)	6	2	4
9. Farm (I)	9	2	7
10. Farm (J)	4	2	2
11. Farm (K)	9	3	6

### 2.3 Data collection

The researchers used various data sources to gather data for the study, including focus group interviews, research memos, and document analysis. The primary source of data collection was semi-structured interviews conducted in focus groups. This approach allowed researchers to ask targeted questions while allowing participants to express their opinions and thoughts on relevant topics (53). The researchers collaborated with all research members involved in the first phase of the study (8) to design the interview guide. They developed 11 questions aimed at enhancing the understanding of the quantitative aspect of the study. The results indicated that employees with less time working on the farm demonstrated less knowledge of the Comprehensive Swine Industry Assessment (CSIA), had a lower perceived ability to identify compromised pigs that required euthanasia, were less willing to perform euthanasia independently, and preferred not to be responsible for informing others about when to euthanize pigs.

Additionally, secondary traumatic stress and transgressions were significantly correlated. These factors were associated with burnout, feelings of betrayal, and overall worker satisfaction. Furthermore, female caretakers reported higher levels of secondary traumatic stress and lower levels of compassion satisfaction. Consequently, the qualitative portion of the study focused on deepening the understanding of euthanasia knowledge, job burnout, stress, satisfaction, and transgressions—defined as actions that conflict with individuals' ethics or beliefs—based on the key findings from the quantitative phase (40).

A total of two days were designated for data collection. To ensure consistency during the focus groups, researchers convened prior to the sessions to read the questions aloud. This allowed them to establish a consistent approach and identify any probes that could be used to clarify the interview guide questions. Furthermore, after the first session participants gathered to share their perspectives and suggest improvements for the data collection process in the second session.

Before beginning the focus groups, the researchers informed the participants about the study's purpose and confidentiality, and participation was voluntary. To ensure anonymity, each participant was assigned a number. The researchers introduced themselves before starting the activity, and with participants' consent, discussions were audio-recorded and transcribed to ensure data accuracy. The interviews lasted between 40 and 80 min. The focus groups were carefully organized to ensure a mix of perspectives. Participants were grouped based on their farm affiliation to facilitate the discussion and to promote building the whole case based on each farm overview. The researchers encouraged open and honest discussions to minimize the effects of socially desirable answers or group influence. They created a comfortable environment where participants could express their views independently. Additionally, the researchers took steps to moderate the sessions to minimize the risk of dominant voices controlling the conversation, ensuring that all participants had an opportunity to contribute equally.

Additionally, the researchers employed “memoing” to capture information that could not be recorded through traditional methods, such as inquiries, comments, personal feelings, and reflections on the interview. Memoing helped increase transparency and reflexivity in the research (58). After each interview and during important moments, the researchers used this method to record observations, personal reflections, and insights that added depth to the qualitative analysis (53). The information collected from the memoing, along with the focus group transcripts and was then matched with document analysis (53). The document analysis involved reviewing relevant written materials, including reports on swine caretakers' job turnover and prior research on similar topics, to ensure data triangulation. Together, these sources provided a comprehensive view of the study's subject, enhancing the validity and depth of the analysis (53, 54).

### 2.4 Data analysis

The participants were asked about their knowledge and preparation for euthanasia, job burnout, stress, satisfaction, and transgressions based on the results from the quantitative phase. To analyze our data, we initially implemented an open coding methodology. As explained by Saldaña (59), this method is particularly useful in case studies because it allows for correcting and redirecting the focus of the analysis. We aimed to deconstruct and reorganize the data by identifying categories that capture the present phenomena. This involved generating categories, subcategories, and dimensions (59). After each person conducted open coding on the transcripts from the focus groups they collected, we met to identify similarities and differences in our coding approaches. Once we reached a consensus on the emerging codes from the data, we moved on to the second coding procedure.

Following this initial stage, we used pattern coding as an explanatory coding approach to creating more meaningful analysis units (59). This data analysis methodology enabled us to connect the data with their sources, including topics, concepts, and themes, and to organize it more flexibly (60). We opted for pattern coding because it facilitates the identification of rules and causes that explain the data. We also grouped the codes into major themes to better understand the phenomena (59). We convened to review the themes that emerged from the data and to reach a consensus among the participants, keeping the discussion open until all coders agreed on the reported information themes.

### 2.5 Qualitative quality

To ensure research rigor in a qualitative study, Creswell and Creswell (53) recommend guaranteeing credibility, transferability, dependability, and confirmability. Credibility refers to the researcher's responsibility to represent the research findings accurately (61). One way to ensure credibility is by providing a profound description and triangulating the data. Our study used multiple data sources, including interviews, research memos, and official online documents, such as reports on swine caretakers' job turnover and previous research conducted with a similar audience.

Transferability corresponds to validity in quantitative research (62). There are several ways to guarantee transferability (53), such as providing a deep description and implementing sampling procedures focused on a specific group. Therefore, we implemented purposive sampling (Refer to participant selection section) to select the participants based on specific characteristics (63).

Dependability, which is comparable to reliability in quantitative research (58), can be ensured by reporting the interviews and explaining how the research was conducted. For our study, we stored the data collection in a dataset that includes interview transcriptions, reflexive memos, and official documents. Additionally, as part of the double-coding procedure, the research team reviewed the information and the codes to ensure agreement on the findings (64).

Finally, we addressed confirmability by practicing research reflexivity, which involves reflecting on our biases and assumptions throughout the data analysis process (61). We used research memos to document and reduce any biases that could have influenced our interpretations, and we frequently reviewed our interviews to identify and address any potential biases.

## 3 Results

### 3.1 Objective 1: understand the effects of euthanizing pigs on Spanish-speaking caretakers

Objective one aimed to explore the potential impacts of euthanasia on Spanish-speaking swine caretakers. Two main themes emerged from the data analysis, stress drivers and burnout drivers among swine caretakers. The stress drivers theme comprised the following subthemes: Ensuring Animal Welfare in Euthanasia, ineffective efforts, and failure to meet production goals. On the other hand, the burnout theme was categorized into two subthemes: Personnel shortage increases workload and inadequate equipment and facilities. The data showed that while the drivers of stress are linked to specific events or failures in the euthanasia process, the drivers of burnout are associated with conditions concerning the general wellbeing and job satisfaction of caregivers. Table 2 describes the themes and subthemes identified for objective one.

**Table 2:** A description of the emerging themes identified for objective one.

**Theme**	**Subtheme**	**Description**
Stress drivers	Ensuring Animal Welfare in Euthanasia	If euthanasia is unsuccessful on the first attempt, the caretaker must administer additional attempts until the pig dies.
	Ineffective efforts	Caretakers may feel like their efforts are ineffective when they treat a pig for an illness, only to find that the pig does not improve and dies or must be euthanized.
	Failure to meet production goals	Due to time constraints and workload, caretakers experience stress if they cannot meet production goals.
Burnout drivers	Personnel shortage increases workload	Staffing shortages impact work-life balance and stress; while some teams adapt positively, heavy workloads persist.
	Equipment and facilities	Workers are frustrated by facility-related challenges, such as inadequate maintenance and tool availability, which lead to stress and inefficiencies.

#### 3.1.1 Theme 1: stress drivers

This theme explores how participants' previous experiences navigate their approach to euthanasia decisions, shaping not only the act itself but also the decision-making process leading up to it. Participants highlighted how past encounters with animal loss, professional pressures, and emotional resilience played a pivotal role in determining their strategies and readiness to address euthanasia. The participants felt great urgency when euthanizing pigs, highlighting the importance of swift and effective action to prevent the animal from further suffering. They emphasized the need for the first attempt to euthanize an animal to be lethal to avoid causing additional pain and having to proceed with a second attempt. However, they also acknowledged the psychological and emotional impact of performing euthanasia and emphasized the importance of ensuring the process is quick and efficient to minimize distress.

Another stress factor for caretakers is the emotional toll of being unable to save sick or injured pigs (otherwise referred to as “compromised”). They expressed deep frustration and helplessness, especially when faced with multiple pig deaths in a brief period. This experience can significantly impact their emotional wellbeing, potentially leading to feelings of isolation.

##### 3.1.1.1 Subtheme 1: ensuring animal welfare in euthanasia

The participants (P) showed a strong desire to euthanize pigs quickly to prevent further suffering, highlighting the importance of taking immediate action. They emphasized the need for effectiveness in the first attempt of the euthanasia process to avoid causing additional pain to the animal with a second attempt. They expressed the importance of minimizing the animal's suffering through a swift and efficient euthanasia process.

Most participants emphasized the need for euthanasia to alleviate animal suffering. They underlined the importance of timely euthanasia to minimize the animal's distress. A participant from Farm H (GH) expressed that “The best decision when identifying a pig that is a candidate for euthanasia is to not leave the animal alive for a single minute, nor a second longer because as more time goes by, the animal suffers” (P3, GH).

During the focus group, most participants highlighted the importance of ensuring that euthanasia is done humanely and effectively. Specifically, in the focus group for Farm F, where all the participants were males, they emphasized the need to guarantee that the initial attempt to euthanize is lethal to prevent any unnecessary pain to the animal that could result from a second attempt. A participant from Farm F (GF) mentioned: “Also, when performing the second attempt [captive bolt euthanasia procedure] is likely more painful for the animal. So, you must make sure that the first attempt is lethal” (P3, GF).

The participants recognized the difficult time that follows the first euthanasia attempt has failed, during which the animal may still suffer. They expressed frustration and stressed the importance of minimizing this suffering by carrying out the euthanasia process quickly and effectively. In Farm B (GB), the participants highlighted the need to use time efficiently. A participant from Farm B (GB) said the following about the critical need for efficient time management during euthanasia procedures: “In the first 5 min [after a failed euthanasia attempt], the animal is in agony, generating frustration (in the caretakers). Then you perform the second attempt at euthanasia; you fail again, then another 5 min, and the same thing happens” (P1, GB).

##### 3.1.1.2 Subtheme 2: ineffective efforts

The study's participants collectively grappled with the emotional strain of caring for pigs. They expressed profound frustration and helplessness when their efforts to treat and assist sick or injured pigs were unsuccessful, resulting in the animals not being saved. They describe the personal stress and frustration that arises when they cannot find solutions for these animal health issues.

Caretakers reflected on the emotional toll of caring for pigs, expressing frustration and helplessness when it still succumbs to illness or injury despite their efforts to treat and assist a pig. A participant from Farm B (GB) expressed the group's feelings and mentioned the following: “Sometimes you can feel frustration when they die. I have come to feel that I could not save her [referring to a sow]. I treated her, helped her, yet I did not save her” (P4, GB).

Participants shared their distress at witnessing multiple pig deaths within a brief time frame. They express a sense of despair and frustration at the helplessness of watching animals die, emphasizing these events' emotional impact on themselves and their colleagues. Researcher memos indicated that each pig caretaker is typically responsible for overseeing up to 1,000 animals, a common practice in large swine production units. This high number may contribute to some caretakers' frustration and feelings of being overwhelmed. A participant from Farm A (GA) said, “They die alone, they die alone. I do not know if some of my co-workers think the same as me, but it causes a lot of despair to see how you take care of the animals, and from one day to the next, you arrive and find 10 or 15 dead animals in a day” (P1, GA).

Farm D participants supported the previous statement and expressed a specific source of stress in their work: the inability to rectify a condition like a prolapse. Prolapse, a condition where an organ slips out of its normal position, can be a significant welfare issue in pigs, often requiring immediate attention (65). They highlight the personal stress and frustration they experience when they cannot solve the animal's ailment, underscoring the emotional challenges faced in their role. A participant from Farm D (GD) expressed the following about the emotional challenges: “So, I get stressed when, for example, a pig is prolapsing, and I cannot correct it” (P5, GD).

Additionally, a participant from Farm J (GJ) mentioned the difficulty in euthanizing animals that are not cooperating with the caretakers: “Yesterday at five, we had to kill one. They killed it because it didn't want to get on the trailer. They spent almost a year trying to get it to the right weight, and they couldn't get it on the trailer, not even with the sows. It would just get to the door, and they couldn't get it to go through. It was a huge animal” (P2, GJ).

##### 3.1.1.3 Subtheme 3: failure to meet production goals

Caretakers described a persistent issue related to productivity, suggesting that they face a daily challenge with their productivity falling into the “red” or below-desired levels. This implies that they often struggle to meet their productivity targets, and this issue is a regular occurrence for them. A participant from Farm G (GG) mentioned the following: “Red numbers in productivity […] Personally, I think about that every day” (P2, GG).

Supporting the information expressed by Farm G (GG) participants, Farm H (GH) participants expressed the frustration of not meeting productivity goals and the constant effort to complete all the tasks planned for the day. They emphasize the struggle to achieve the intended objectives and the stress of falling short of these targets. One of the participants in Farm H (GH) expressed the following: “Not getting there by not hitting the numbers, trying to finish everything that had been planned for the day stresses you” (P3, GH). Additionally, similarly to the previous comment, another participant from Farm H (GH) discussed the stress that arises when there is a buildup of numerous tasks or activities that need to be completed. They highlight the pressure of dealing with this accumulation of responsibilities and the associated stress in trying to accomplish them. The participant stated: “When you have many activities piled up, activities that you have to make happen, you get stressed because you have to make them happen” (P5, GH).

Finally, the same focus group from Farm H (GH) mentioned the stress related to managing their time efficiently while monitoring the wellbeing of sows during farrowing. They said they feel pressured to ensure that sows are checked on, identify issues, and maintain the correct timing for various tasks, mainly when there are many sows in the process of giving birth. This can occasionally lead to stress. A participant from Farm H (GH) mentioned the following: “Well, if you are stressed, you must still manage your time to check your sows and ensure they are farrowing well. You must see which ones have a problem, which causes a little stress. It is not a lot, but yes, sometimes you have stress when you have a lot of open/synchronized births, and you must check the sows with your timings right” (P1, GH).

#### 3.1.2 Theme 2: burnout drivers

##### 3.1.2.1 Subtheme 1: personnel shortage increases workload

Swine caretakers experience burnout and stressful situations in their job roles. Some drivers of this emotional status are the extra workloads and the lack of personnel to help achieve the work. Two participants from Farm J (GJ) mentioned the following: “I think that in this case, what is worrying is the lack of staff” (P2, GJ), and “Sometimes you do not just do your job, you also have to do your partner's job” (P4, GJ), meaning that swine caretakers are aware that if there are not enough workers to perform the activities, they must complete the extra workload. Additionally, a participant from Farm B (GB) corroborated the previous statements by saying that “When there is a lack of workers, then there is a lot more work to do, and it stresses the team a lot” (P9, GB).

Understandably, the situations mentioned above could occur in different conditions; as a participant from Farm K (GK) said, “They (situations) can be unforeseen events that change the plan of the day, and you have to change everything again, the whole plan. If unforeseen, they can vary from the fact that there are no people. If something broke down, you must fix it” (P2, GK). Another participant emphasized that while euthanasia could be a choice, it is often a necessity. He explained: “There are people who decide not to do it or who do not want to do it, but in the end, there are situations where you find yourself alone, and well, you have to do it because I think it is even worse to leave the animal suffering.”

##### 3.1.2.2 Subtheme 2: equipment and facilities

Some other essential topics discussed during the focus groups pertained to how stressful the lack of necessary equipment and maintenance of the facilities can be. A caretaker from the Farm I (GI) mentioned, “The lack of maintenance to the facility is stressful because the work is complicated as it is, and I do not have much patience, although maybe I do not know if it is noticed or not, but I stress about this kind of thing, and I get in a bad mood” (P3, GI). This previous quote highlights the importance of an adequate facility for the employees to reduce the probability of experiencing stress during work activities. The same participant extended the previous comment by stating: “Yes, it is stressful[…] Not having the tools or the right place to accommodate the animals in a good place stresses us all and prevents us from achieving some objectives, right? (P3, GI).

It is important to recognize that equipment and facilities vary depending on the farm; the participants had diverse opinions, and this is related to the farm in which they are currently employed; for example, a participant in Farm A (GA) mentioned:

“The facility maintenance is stressful. I was on another farm where you were walking, and suddenly, a pig got out and headed toward you, and 20 other pigs followed from the open pen. You lose and waste so much time getting things done again. Moreover, the worst thing is that you must do it every day because the same thing happens every day. They do not invest in effective maintenance of the facilities. I had to work on a farm where we had to move the same pigs every day, and suddenly, I had to use garbage bags, ties, wires, chains, and whatever I could to keep the pigs inside their pen. Now for example, the problem is less at this farm but, we currently have a problem with the washing pumps that are always failing, which causes delays, delaying the entire cycle of work and production” (P10, GA).

### 3.2 Objective 2: to explore how caretakers' experiences affect Euthanasia decision making

Objective two aimed to understand the experiences that could affect performing euthanasia decision-making. Three themes emerged from the data: (1) Euthanasia becomes easier with practice, (2) Knowledge and Education, and (3) Cultural Background. Table 3 describes the emerging themes identified for the second objective.

**Table 3:** A description of the emerging themes identified for objective two.

**Theme**	**Description**
Euthanasia becomes easier with practice	Euthanasia is a challenging process, but caretakers can perform it more easily with proper training and repetition.
Knowledge and education	Learning about pig farming takes time and experience, including education in euthanasia, is critical. The type of education a caretaker receives depends on their role and job responsibilities.
Cultural background	Participants come from different backgrounds with a wide variety of experiences, which could influence their decision-making in performing euthanasia.

#### 3.2.1 Theme 1: Euthanasia becomes easier with practice

This theme highlights the initial nervousness and self-doubt individuals may experience when facing new tasks or processes, such as adult pig euthanasia. Participants expressed that the first encounters with such responsibilities can be daunting, and adapting to new situations can cause feelings of anxiety or worry. One of the participants from Farm G (GG) expressed: “The first time [euthanizing], there is always nervousness” (P2, GG).

Nevertheless, it is possible to overcome such feelings with time and practice. A participant from Farm K (GK) noted, “I believe that not having previous experience could make people doubt if they are actually doing something good or not, which could be most people's major concerns. As you gain practice [regarding euthanasia], that goes away [nervousness]” (P9, GK). Her comment suggests that the more familiar they become with a particular task or process, the less anxious they may feel, and that it is common for individuals to experience self-doubt when they lack prior experience in each area.

Experience is crucial in shaping one's perspective toward a task or process. As a participant from Farm E (GE) emphasized, “I think it is more about the experience. Because I have seen people who have worked in pig production in other places, and it impacts them, it's not something completely new, right? On the other hand, I know people who focus on clinical work, and when you talk about the process, they suggest suing the company, because they do know the process. So, I think it depends on one's prior experience or how they personally respond to euthanizing animals”' (P5, GE). With the right mindset and support, individuals can overcome nervousness and self-doubt, gain confidence, and become more adept at handling new challenges. For example, a participant from Farm F stated, “I think positively because every time we perform a procedure wrongly, we improve the next time” (P7, GF).

#### 3.2.2 Theme 2: knowledge and education

The education and prior knowledge that swine caretakers possess regarding swine euthanasia significantly influence their decision-making and the timely execution of euthanasia. When asked how they would feel better prepared to perform euthanasia on pigs, a participant from Farm F (GF) shared, “It is important to know about both areas, farrowing and breeding, because sometimes we tend to both areas, it is like we are more balanced like we have a broader perspective and we realize, yes, it is very vital to implement euthanasia in both areas” (P6, GF).

Many participants in the study had some form of education in veterinary sciences. A participant from Farm H (GH) shared, “The majority of us have already seen these topics in college; you get involved in this topic [euthanasia], and by the time you do it at work, it seems very normal” (P6, GH). Another participant in the same focus group recalled, “As students, we practiced in slaughterhouses” (P3, GH). A focus group F (GF) participant supported this claim and added, “Well, almost all of us have started dealing with it since we entered university to study for our bachelor's degree. You start becoming sensitized. From the moment you enter, you know you are going to see blood, surgeries, and you will be dealing with life and death” (P1, GF).

Participants also recognized that caretakers without a veterinary educational background find euthanasia difficult. A participant from Farm H (GH) explained, “People who have not studied veterinary medicine, such as some agronomists I have met on other farms, may at first not want to euthanize” (P2, GH). It is important to note that some caretakers had their first experience with euthanasia in their current job, as one participant from Farm J (GJ) shared, “I came here and got to know about animal euthanasia” (P2, GJ). Even those who had prior knowledge found the experience different. As another participant from Farm J (GJ) noted, “Well, it is just that I really started doing it, here as well. I mean, yes, we obviously knew about euthanasia, we knew the methods” (P3, GJ). Moreover, a participant from Farm G (GG) noted, “Not all of us who came to the company are veterinarians, and even among veterinarians, not all of us specialized in pigs” (P6, GG).

Despite the challenges, many participants recognized the importance of euthanasia regarding animal welfare. As a participant from Farm K (GK) explained, “I do not enjoy doing it [euthanasia], but we do it because the suffering will be over, mainly because then we understand, we comprehend that it is not for pleasure but because it has to be done” (P2, GK).

#### 3.2.3 Theme 3: cultural background

This study focuses on Spanish-speaking swine caretakers from Latin America, particularly Mexico. Acknowledging that the following quotes refer to past experiences in their home countries, highlighting cultural differences is essential. As one participant from Farm F (GF) mentioned: “I previously worked in a beef slaughterhouse and also a municipal slaughterhouse, you could say where there were beef cattle and pigs. There, the method of euthanasia was completely different, where animals really suffered” (P3, GF). Another participant from Farm K (GK) added, “It could be a bit more grotesque in a ranch or in a place different from what we do here” (P14, GK).

Some participants grew up in rural areas where slaughtering animals was a common practicel. As a caretaker from Farm G (GG) mentioned, “My family is from the countryside, and obviously, as I come from there, euthanasia does not make me sad” (P5, GG). Another participant from Farm K (GK) added, “I saw it on ranches, how they slaughtered animals, but it did not affect me for the same reason that I'm used to weapons, to killing chickens for meals. I came here with that experience of killing little animals” (P9, GK).

Furthermore, for some participants, performing euthanasia is a part of their cultural tradition. As one participant from Farm G (GG) expressed, “For me, it is something [euthanasia] that seems very normal in itself, [a part of life]. It is like the tradition of barbecuing, it is a tradition that all women make the barbeque, we all have to know” (P6, GG). These quotes highlight the importance of understanding the cultural differences among swine caretakers from various backgrounds and the need for cultural sensitivity training to promote proper euthanasia practices.

Finally, participants stressed the importance of recognizing cultural differences and strengthening cultural competencies, as they have encountered situations where managers showed preference to caretakers from the United States. A participant from Farm J (GJ) echoed this sentiment:

“For example, there are American and Mexican workers on this farm. I think the company could strengthen its approach by training the supervisors more. They should manage with the same standards for both [international Hispanic workers] us and them [American workers]. Sometimes there is a noticeable difference. For instance, there are situations where you might do something wrong, maybe due to a lack of training or some other reason. And perhaps you face more severe consequences than someone who is a citizen. Also, there are American supervisors who sometimes literally spend their time not doing much, and nothing happens to them, no one says anything. But in our case, no, they do call us out. I feel that the company tries not to favor any side, but obviously, it relies on the judgment of those who are already supervisors, right? So that's where I think the problem lies. For example, when we, who are just starting to climb the ranks, have problems and communicate them to our supervisors, it seems like they are already biased toward certain people. And then the company, without knowing what's going on, asks the supervisors, and the supervisors say, ‘Hey, that's not how it is.”'

### 3.3 Objective 3: to understand how caretakers could feel more prepared to perform Euthanasia

For objective three, participants in the focus groups state some potential training topics to improve caretakers' wellbeing, making them feel more prepared to perform euthanasia. Three themes emerged from the coding process: (1) Safety Concerns, (2) Confidence, (3) Managing Emotions and Therapy. A description of the themes is provided in Table 4.

**Table 4:** A description of the emerging themes identified for objective three.

**Theme**	**Description**
Safety concerns	Euthanasia is a process that could bring some fearful feelings for caretakers in terms of having an accident due to the inappropriate condition of the equipment or making bad use of it.
Confidence	Experience and confidence in the euthanasia process are closely related because participants mentioned that some workers are more confident if they have previous experience; therefore, even if workers get the knowledge from training, it is also important to work on performance proficiency.
Managing emotions and therapy	This theme emerged in most focus groups because to decide to euthanize, it is important to know how to manage emotions and consider implementing thanatology therapy to support workers in handling negative feelings regarding the process.

#### 3.3.1 Theme 1: safety concerns

Safety concerns emerged prominently, particularly in the euthanasia process for both adult pigs and young pigs. This theme encapsulates the workers' apprehension and the precautions they take when dealing with euthanasia of animals, especially when it comes to sows in poor health.

Euthanasia was performed on adult and young pigs, with young pigs being euthanized more frequently. As an example, when caretakers are required to euthanize a sow that is sick or in bad condition, they need to put more effort into performing this activity; a participant from Farm A (GA) mentioned: “I don't think it's a matter of difficulty on our part. We've done it on several occasions, but yes, it might be a bit riskier with an adult sow because it's stronger than piglets, and we need to use more force to hold it. The sow often reacts in a way that reflects its response after receiving the blow with the captive bolt. It's not the blow but the perforation. Usually, the reflex of the sow is to lift its head, and if you don't have it really well secured, you could injure yourself” (P1, GA).

Participants emphasize the critical role of proper equipment in ensuring safety. They stress that not only does the equipment need to be suitable for the task at hand, but it must also be in conditions to avoid potential harm to the animals and the workers themselves. One participant from Farm K (GK) mentioned the importance of having good equipment and not risking worker safety. He expressed: “It is necessary not only for the method to be executed properly but also for your own safety that all the equipment you will use is in the best condition. However, it doesn't guarantee that it will work perfectly 100% of the time. Sometimes, something will be different. They are animals, and they are not the same. Sometimes, despite applying everything correctly, the method of euthanasia may not be effective. And sometimes, you need to notify the managers for a solution. We've done this before, as we proceed, we need to call and see what managers suggest because sometimes things get complicated” (P9, GK).

Finally, participants expressed “fear” as another salient component within this theme. Workers openly express their concerns about potential injuries and accidents during the euthanasia process. Some participants feel uneasy about the physical demands and the inherent risks involved in this aspect of their work. As a result, some workers may opt to include others in the procedure, prioritizing their safety and emotional comfort. A participant from Farm J (GJ) expressed the feeling that other groups had regarding fear. He said: “I think it is fear of getting hurt” (P4, GJ), supporting the previous comment From Farm A (GA). Caretakers expressed their fear of manipulating the tools. One of the participants mentioned: “It scares me. I had some difficulty in firing the gun and preferred to tell another person to do it” (P1, GA).

#### 3.3.2 Theme 2: confidence

Confidence plays a pivotal role in euthanasia procedures on the farm. Focus group participants emphasized that confidence is crucial for performing euthanasia effectively and humanely.

A key factor highlighted by most participants is the importance of confidence during the euthanasia process. They believe confidence arises from trusting one's training and abilities. While training is valuable, it alone is not sufficient. As one participant from Farm K (GK) noted, “Trust [in ourselves]. It could be the training, but we already have it. We need just trust” (P9, GK). Supporting this view, participants from Farm G (GG) emphasized that beyond acquiring the necessary skills, individuals must also trust themselves and their competence to perform euthanasia effectively, recognizing the benefits of practicing to lead to proficiency. One participant shared, “From my point of view, it's all psychological. The same thing happened to me with a guy who went to training and was afraid of euthanizing. I told him that, at least for me, it's more upsetting to see the little piglet so small and suffering.”

Preparation is also crucial for building confidence. Participants voiced concerns about the adequacy of their training and prior experience. They acknowledged that not all farm caretakers have veterinary backgrounds or experience with pigs. A participant from Farm K (GK) expressed worries about making mistakes during the euthanasia process, potentially causing harm rather than relief. The participant stated, “I would think that the most common concern would be not doing it correctly. I don't have experience. Maybe I'll do it wrong and create a bigger problem instead of relieving it. Or maybe that's the biggest worry for many people, because I start thinking I'm being pessimistic. It may go wrong, or it could end up going better [than I thought]. So, as you gain practice, this happens; perhaps that's the initial fear” (P9, GK).

A participant from Farm J (GJ) emphasized the importance of training caretakers to promote their confidence. He expressed, “It's about having the confidence to do it. When new people arrive at the farm, then you gradually teach them. First, you do it and let them watch, and then little by little, you let them do it.”

#### 3.3.3 Theme 3: managing emotions and therapy

The theme of emotion management and therapy was a critical aspect of discussions surrounding euthanasia processes. This theme underscores the emotional complexities that individuals experience when making euthanasia decisions and performing the procedure.

Managing emotions emerged as a significant factor in this theme. Participants recognize the importance of being emotionally intelligent to make sound decisions regarding euthanasia. They noted that a common factor among those who struggle with performing euthanasia is the trauma that can develop afterward. A participant from Farm K (GK) explained: “There are situations, maybe in the worst-case scenario, where an inexperienced person is [performing the procedure] and it goes wrong. I believe that can really affect them. Maybe someone sensitive does it and then doesn't want to do it again because they see the disaster that occurred. How do we eliminate that bad experience for people, so they regain confidence or aren't affected? That is an interesting topic. If a worker calls me and says, 'I had this experience, I got home and still feel anxious, thinking I did it wrong, and I don't want to do it again,' how to handle that case would be an interesting topic” (P9, GK).

Participants acknowledged that the process often involves emotions and feelings that can influence decision-making and the execution of euthanasia. This emotional dimension can be particularly challenging for more sensitive individuals. Therefore, addressing the psychological aspects and traumas caused by performing euthanasia is essential. The term thanatology emerged from the data, referring to the study of death and dying (66). Participants suggested that a thanatological approach could help individuals assimilate the inevitability of death and potentially reduce the emotional trauma associated with euthanizing animals. For example, one participant from Farm G expressed: “Thanatology helps you a lot in coming to terms with the death of someone, which is inevitable” (P2, GG).

The need for emotional therapy is a notable component of this theme. Participants suggested that individuals who struggle with the emotional aspects of euthanasia, including feelings of guilt and sorrow, should have access to emotional therapy. Therapy could provide support and coping mechanisms for those who find the euthanasia process emotionally taxing. This claim was expressed by a participant from Farm K (GK), who stated: “There should be emotional therapy for people who are more sensitive when it comes to deciding [to euthanize] because often people feel guilty, thinking it is their fault that they are going to kill the animal. Sometimes, they might think that, but really, it is a good. I see it from the perspective that it is not so much about killing an animal but ending the problem the animal has. Maybe some people can't see it that way and may react in the opposite manner, saying they don't want to kill an animal” (P8, GK).

Another participant in the same group supported this claim and expressed: “In my case, it was more emotional because I had been doing it for 3 years and suddenly wasn't anymore. I think it also affects how you feel emotionally; even hormones play a big role. I remember when there was another person here who no longer works here; she could do it normally, but when she was pregnant, she stopped doing it, and someone else had to do it” (P1, GK).

## 4 Discussion

For objective one, the predominant themes identified from the data were caretaker stress and burnout. While work stress and burnout are related, they are distinct concepts. Work stress arises from adverse workplace conditions, whereas burnout is a state of physical, emotional, and mental exhaustion due to prolonged exposure to work stress (67–69). The data showed that stress drivers are linked to specific events or failures in the euthanasia process. These factors create immediate stress for caretakers as they deal with these challenges. On the other hand, burnout drivers contribute to long-term exhaustion and disengagement because they represent ongoing stresses that affect caregivers' ability to perform their duties effectively over time.

The importance of animal welfare on farms has been highlighted by various researchers (3, 70, 71, 82). However, the mental health of caretakers and the potential effects of stress on them have often been overlooked. This study examines workload and equipment failure as significant burnout factors. Similar findings were reported by Rabinowitz et al. (72) and Newsome et al. (69), who found that caretakers working closely with animals experience higher levels of fatigue, leading to increased stress, burnout, and high job turnover (73, 79).

Participants recognized the stress of constantly trying to help animals recover and then ending up having to euthanize them due to unsuccessful recovery. One of the reasons for the stress could be the “caring-killing paradox,” which has been explored in research on animal shelter workers tasked with euthanizing companion animals they were caring for (74). Both compassion fatigue and the caring-killing paradox have been suggested as factors affecting euthanasia decisions on swine farms (3).

The findings of this study align with previous research highlighting the importance of proper training, equipment, and working conditions in preventing injuries and accidents among animal handlers (86). Anderson et al. (75) further emphasizes the need to consider animal size when performing euthanasia, reinforcing the necessity for tailored techniques and specialized equipment. Addressing these factors and adopting best practices can enhance euthanasia procedures, reducing suffering for both animals and caretakers. Additionally, participants noted that equipment issues, such as malfunctioning washing pumps and poor facility maintenance, often hinder their ability to manage pigs effectively. These failures lead to delays in euthanasia, which in turn affect the timeliness of the procedure and negatively impact animal welfare, causing unnecessary suffering for the animals and increasing stress for the caretakers (11).

Participants noted stress from the prolonged suffering of pigs until their death, futile efforts, and failure to meet production goals. Andrukonis and Protopova (40) found similar information in their research on animal shelter employee wellbeing and how the euthanasia process could cause moral injury to caretakers. Norman and Maguen (42) also concluded that moral damage could lead to post-traumatic stress disorders, depression, and other disorders, where feelings such as guilt, shame, betrayal, and anger are predominant, even without a formal diagnosis. The decision to perform euthanasia can be difficult for animal caretakers, and their experiences can influence decision-making.

For objective two, to explore how caretakers' experiences affect performing euthanasia decision-making, three key factors were identified: frequency, knowledge and education, and cultural background. Caretakers who perform euthanasia more frequently tend to find the process more manageable over time. This is consistent with the concept of desensitization, where repeated exposure to a stimulus reduces its emotional impact (76). Caretakers without prior experience may feel nervous and self-doubtful. However, as they practice more, they gain confidence and are less affected by the process. This finding is consistent with Edwards-Callaway et al. (16), who also highlighted that experience reduces emotional distress. Similarly, Campler et al. (77) emphasized the importance of interactive training programs, which equip caretakers with the skills necessary to build confidence more quickly. These studies suggest that proper training can better prepare caretakers, ensuring they possess the necessary knowledge and abilities to handle euthanasia decisions more effectively.

Knowledge and education are critical to performing euthanasia effectively. Participants widely mentioned that education in veterinary medicine and related sciences helped them understand the rationale behind euthanasia and complete it more confidently. They do not enjoy euthanizing but recognize its importance in mitigating compromised animal welfare when recovery prospects are low (69). As Acevedo et al. (8) mentioned, certain traits of caretakers, like their educational background, years of experience in the swine industry, and duration of employment on the farm, can impact how euthanasia practices are carried out in the swine industry.

Cultural background plays a significant role in shaping how individuals perceive and manage euthanasia. Participants in this study noted that those with prior exposure to animal slaughter or euthanasia in their home countries often experience less trauma during the process. However, cultural differences, combined with tensions between native and foreign caretakers, were reported to contribute to workplace stress. Mullins et al. (3) further emphasize that cultural factors can hinder timely euthanasia decisions, highlighting the importance of understanding caretakers' diverse backgrounds to enhance the success of euthanasia programs.

Educational background is another critical consideration. Edwards-Callaway et al. (16) found, similar to our findings, that training programs must be tailored to match caretakers' educational experiences. Many caretakers bring formal knowledge from previous roles, which can be leveraged to support those with less expertise in animal care. By customizing training based on educational background, caretakers of all levels can be better equipped to perform euthanasia effectively.

For objective three, the main findings highlighted safety concerns, which align with previous research emphasizing the importance of proper training, equipment, and working conditions in preventing injuries and accidents among animal handlers. In support of this finding, Walker et al. (14) highlighted that there are challenges related to performing euthanasia promptly, such as inconsistencies in treatment protocols and insufficient employee training. Also, Mullins et al. (3) mentioned that the physical location and condition of equipment are obstacles to performing euthanasia promptly.

The relationship between confidence and experience in euthanasia is consistent with previous research on skill acquisition and performance (3, 8). Hartnack et al. (9) reported that veterinarians were more likely to disagree with euthanasia in some scenarios. Similarly, this study revealed the new caretakers' disagreement, fear, and lack of confidence. However, unlike Hartnack et al. (9), this research highlights specific concerns of caretakers related to the adequacy of training and the need for ongoing support and development.

The findings on the emotional impact of euthanasia and the importance of emotional support align with previous research on the psychological wellbeing of animal handlers. For example, as Walker et al. (14) mentioned, euthanasia decisions have a profound impact on animal caregivers, in some cases causing “moral stress,” which leads to a variety of health problems, both psychological, and emotional. As a result, this study supports these statements. It adds to the literature significant findings by emphasizing the specific needs of caretakers in agricultural settings and the potential benefits of thanatology-based interventions.

## 5 Conclusions

This study has provided insights into the multifaceted experiences of Spanish-speaking swine caretakers and their decision-making processes regarding euthanasia. Key themes identified include stress, burnout, compassion fatigue, and the complexities of the caring-killing paradox. These findings carry significant implications for the wellbeing of swine caretakers and the welfare of the animals under their care.

This study underscores the importance of proper training and emotional support for swine caretakers. While technical proficiency is essential, addressing the psychological impact of euthanasia is equally critical. Providing mental health resources, such as emotional therapy, can support caretakers struggling with the emotional aspects of euthanasia. Addressing guilt and sorrow is vital for their wellbeing and work performance.

Recognizing and addressing cultural differences in euthanasia decision-making is essential. Training programs should incorporate cultural sensitivity and the importance of humane practices. In addition to the training provided to swine caretakers on the farms, our study, although focused on Spanish-speaking caretakers, yields insights that may be relevant to other groups involved in livestock production. The themes that emerged from our focus groups encompass stress, burnout, compassion fatigue, the intricacies of decision-making, and hold the potential to educate the swine industry about the need of TN Visa workers and the Hispanic workforce to improve the wellbeing of caretakers across diverse backgrounds. This integrated approach benefits caretakers, the swine industry, and contributes to enhancing sustainability by upholding animal welfare standards, and ensuring the psychological safety of those entrusted with the care of animals (85).

## 6 Recommendations

Swine caretakers' feedback suggests that to improve the euthanasia process, a more comprehensive training program should be implemented. This program should ensure that individuals understand the necessity of prompt euthanasia for certain animals and achieve proficiency in the procedure. The training could begin with the use of animal models, followed by practice with cadavers, observation of an experienced professional, supervised practice, and, finally, independent performance of euthanasia. To implement this training effectively, a phased, hands-on training model should be created, starting with initial training modules where caretakers practice on animal models and cadavers to simulate real-life conditions. This should be followed by supervised practice, where caretakers observe experienced professionals performing euthanasia procedures and then perform euthanasia under supervision, ensuring proficiency before progressing to independent performance.

In addition to technical training, program implementers and evaluators should incorporate components that address stress and burnout, emphasize the reasoning behind euthanasia decisions, and account for cultural differences. The training should be adaptable to different education levels and ensure that all participants develop proficiency in humane euthanasia methods. Furthermore, establishing mental health support systems for caretakers is essential, with regular check-ins, therapy options, and resources to help workers manage the emotional strain of performing euthanasia. Developing programs that focus on emotional intelligence and thanatology can also aid caretakers in coping with these challenges.

Investing in training that enhances emotional intelligence can help caretakers navigate the emotional complexities of euthanasia, improving both their decision-making and overall wellbeing. Farms should also implement strategies to manage non-cooperative animals during euthanasia, as handling difficulties can negatively impact both animal welfare and operational efficiency. Specialized techniques and equipment should be used to address these challenges, ensuring smoother procedures and minimizing stress for both animals and caretakers.

Additionally, creating a supportive environment is essential for reducing stress and burnout. Regular group meetings where workers can share their experiences, express concerns, and receive guidance on emotional distress should be established. Open communication channels should be implemented to allow workers to report concerns about the euthanasia process, equipment, or emotional challenges without fear of retribution.

Training programs should also be tailored to accommodate the varying levels of agricultural experience between local U.S. workers and TN visa workers. Customized training modules should be created to ensure that all workers, regardless of their background, receive guidance that is specific to their level of experience. Cultural sensitivity training should also be included to ensure that workers are comfortable and confident with euthanasia procedures. To better understand the workforce's needs, workforce surveys should be conducted before and after training to gather feedback on the effectiveness of the program, emotional wellbeing, and equipment issues. Regular satisfaction surveys should also be implemented to assess ongoing challenges faced by workers, including emotional strain, equipment malfunctions, or difficulties with the euthanasia process. By incorporating these concrete steps, training programs will be more effective in improving both the proficiency of swine caretakers and their emotional wellbeing, ensuring humane treatment of animals and fostering a supportive and efficient work environment.

Lastly, it is critical that training includes troubleshooting scenarios so caretakers understand how to handle various challenges that may arise. Worker safety should also be a central focus, with basic safety information covering the proper use of equipment and the ability to recognize equipment malfunctions. Ensuring that workers are well-equipped to prevent injury to themselves and avoid using faulty equipment on animals will further improve both the safety and effectiveness of euthanasia procedures.

## 7 Limitations

This study relied on self-reported data collected through focus groups. This approach may introduce limitations related to social desirability bias where participants may adjust their responses to align with perceived expectations or to avoid discussing potentially stigmatized aspects of euthanasia-related tasks. This could influence the authenticity of responses, particularly on sensitive topics such as emotional distress and burnout.

Additionally, while the study addresses stress and burnout, the psychological assessment was limited in scope, relying on self-reported qualitative insights rather than standardized mental health evaluations. Incorporating formal psychological measures could provide a more comprehensive understanding of the mental health impacts, enabling comparisons with broader occupational stress data.

Another potential limitation of this study is the organization of focus groups by farm, which may have contributed to social desirability bias. Participants who work closely together might have felt pressured to provide responses that align with their peers' expectations or the perceived norms of their workplace. While this approach allowed us to capture farm-specific contexts and realities, it may have constrained the openness of some participants' contributions. Future studies could consider mixing participants from different farms in focus groups. This strategy might help reduce the influence of social dynamics within familiar groups and encourage more candid and diverse perspectives, ultimately enriching the data.

Finally, as a cross-sectional study, the data capture a single point in time, limiting a better understanding of the potential evolution of stress and burnout among caretakers. A longitudinal study would allow for observing changes over time, especially in response to varying farm conditions, personnel shifts, and evolving practices, providing a clearer perspective on the long-term psychological effects and coping strategies among swine caretakers.

## Data Availability

The original contributions presented in the study are included in the article/supplementary material. The data is available through Texas Tech School of Veterinary Medicine. Further inquiries can be directed to the corresponding author.
